# Antitumor potential of a synthetic interferon-alpha/PLGF-2 positive charge peptide hybrid molecule in pancreatic cancer cells

**DOI:** 10.1038/srep16975

**Published:** 2015-11-20

**Authors:** Hongmei Yin, Naifei Chen, Rui Guo, Hong Wang, Wei Li, Guanjun Wang, Jiuwei Cui, Haofan Jin, Ji-Fan Hu

**Affiliations:** 1 Stem Cell and Cancer Center, First Hospital, Jilin University, Changchun, Jilin 130021, China; 2 Stanford University Medical School, Palo Alto Veterans Institute for Research, Palo Alto, CA 94304, USA

## Abstract

Pancreatic cancer is the most aggressive malignant disease, ranking as the fourth leading cause of cancer-related death among men and women in the United States. Interferon alpha (IFNα) has been used to treat pancreatic cancer, but its clinical application has been significantly hindered due to the low antitumor activity. We used a “cDNA in-frame fragment library” screening approach to identify short peptides that potentiate the antitumor activity of interferons. A short positively charged peptide derived from the C-terminus of placental growth factor-2 (PLGF-2) was selected to enhance the activity of IFNα. For this, we constructed a synthetic interferon hybrid molecule (SIFα) by fusing the positively charged PLGF-2 peptide to the C-terminus of the human IFNα. Using human pancreatic cell lines (ASPC and CFPAC1) as a model system, we found that SIFα exhibited a significantly higher activity than did the wild-type IFNα in inhibiting the tumor cell growth. The enhanced activity of the synthetic SIFα was associated with the activation of interferon pathway target genes and the increased binding of cell membrane receptor. This study demonstrates the potential of a synthetic SIFα as a novel antitumor agent.

Pancreatic cancer is the fourth leading cause of cancer-associated death, being responsible for 7% of all cancer-related deaths in both men and women^1^,^2^. Currently, no effective therapeutic regimens are able to significantly ameliorate the progress of the disease. The prognosis of pancreatic cancer is poor, with the 5-year survival rate ∼7%. Until now, surgery is the only curative therapy. However, most pancreatic cancer patients are diagnosed at the advanced stage. As a result, only about 10 ∼ 20% of patients are considered candidate for surgery^3^. Chemotherapy is widely used as the main therapeutic approach in the treatment of pancreatic cancer. However, the most effective chemotherapy regimens can only prolong overall survival by several months^4^,^5^, primarily due to the chemo/radio-resistant behavior of pancreatic cancer cells. Therefore, it is urgent to develop novel therapeutic strategies to prolong the survival of the disease.

Recently, accumulating evidence shows that IFNα, a natural potent pleiotropic cytokine, has antitumor effect and restitutes the chemosensitivity in pancreatic cancer and other solid tumors^6^,^7^,^8^. However, the potency of IFNα therapy is significantly limited by its systemic toxicity^9^,^10^. Long-term parental administration of IFNα is required to maintain therapeutic efficacy, and this often induces high-grade toxicity and significant side effects in many patients.

To potentiate the antitumor effect of interferon, we developed a “cDNA in-frame fragment” library screening technology. In this approach, a random library of short double-strand cDNA fragments was fused “in frame” to the C-terminus of IFN. By screening, we identified short cDNA fragments that enhance the activity of IFN (“IFN enhancer peptide”, IEP). Interestingly, three IEP peptides contain a short stretch of positively charged amino acids derived from placental growth factor-2 (PLGF-2)(Guo *et al.* unpublished data). This short peptide has been shown to enhance the activity of three growth factors (vascular endothelial growth factor-A, platelet-derived growth factor-BB, and bone morphogenetic protein-2)^11^.

In this proof-of-concept study, we examined whether this novel IEP peptide was able to potentiate the antitumor activity of IFNα. We determined whether a synthetic IFNα-IEP fusion protein, when delivered by a lentiviral vector, was able to enhance the inhibition of cancer cell proliferation and invasion. At the same time, we also examined whether the synthetic interferon was able to modulate the effect of the chemotherapeutic drug gemcitabin (GEM) in human pancreatic cell lines.

## Materials and Methods

### Cell culture

Pancreatic cancer cell line ASPC was purchased from the American Type Culture Collection (ATCC, VA) and CFPAC1 was obtained from Dr. Julien Sage, Stanford University School of Medicine^12^. Both cells were routinely cultivated in DMEM medium (Invitrogen, CA), supplemented with 10% fetal bovine serum (Invitrogen, CA), 100 U/ml penicillin and 100 μg/ml streptomycin at 37 °C in a humidified atmosphere containing 5% CO2.

The lentiviral packaging 293T cells were purchased from ATCC (Manassas, VA) and cultured in DMEM supplemented with 10% FBS, 1x Non-Essential Amino Acid (NEAA), and 100 U/ml Penicillin-Streptomycin (Invitrogen, CA).

### Library screening of interferon-enhancer peptides

**Figure 1:**
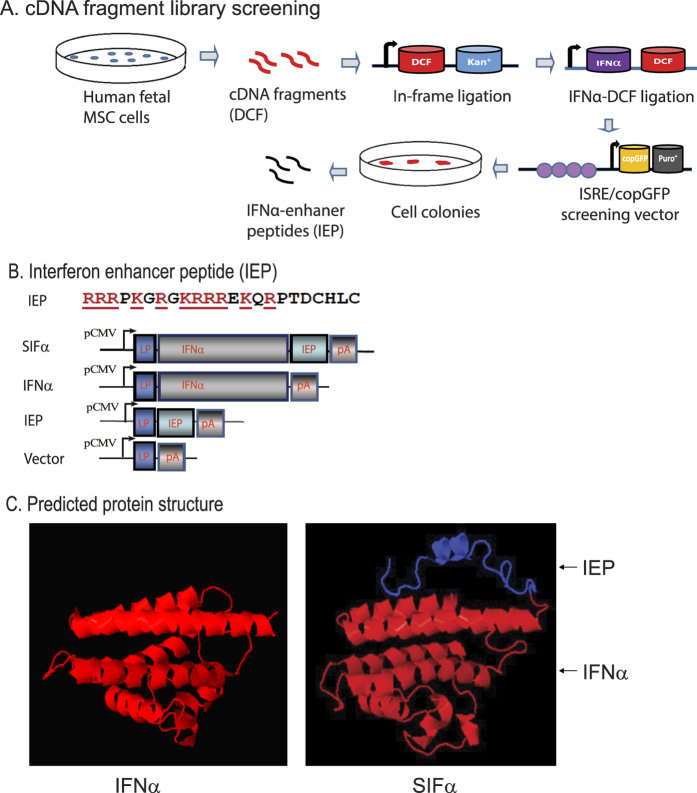
Synthesis of the interferon SIFα-IEP hybrid protein. (**A**) Schematic diagram of “cDNA in frame fragment” library screening of interferon-enhanced peptides. Double-strand cDNAs from IFN-responding cells are sonicated. The gel-purified short fragments are ligated in frame with translation initiation code “ATG” of kanamycin. The “ATG-DCF-Kan+ colonies are selected by kanamycin and the “in-frame” DCFs are digested by BamH1/EcoRV and are ligated “in frame” to the C-terminus of IFN. The IFN-DCF lentiviral library is used to transfect 293T cells that carry the ISRE/copGFP/Puro+ reporter. The IFN-enhancer peptides (IEP) are recovered from cell clones that are resistant to puromycin and have the strong fluorescence of copGFP reporter. (**B**) Synthetic Interferon alpha (SIFα) vector. IEP: the consensus IFN-enhancer peptide. The positively charged amino acids are labeled in underlined red, where R = arginine, K = lysine. pCMV: CMV promoter; IFNα: wild type interferon alpha; SIFα: synthetic interferon alpha (IFNα-IEP); IEP vector: the control construct that contains only the IEP peptide; vector: vector control. (**C**) Predicted protein structures of IFNα (left panel) and the synthetic SIFα (right panel). The positively charged IEP peptide is shown in blue on the top of the IFNα structure. The putative structure of SIFα was predicted using the software on the website: http://zhanglab.ccmb.med.umich.edu.

Interferon-enhancer peptides were identified by “cDNA in-frame fragment library” screening (Fig. 1A). In this approach, the IFN-enhancer peptides (IEPs) were screened by fusing the short in-frame cDNA fragments with IFNα. For convenience, the random short cDNA fragments were generated from mRNAs isolated from human fetal heart mesenchymal stem cell-derived fibroblast like cells^13^. Specifically, mRNAs were isolated from fibroblasts using the Dynabeads® mRNA DIRECT kit (Invitrogen, CA) and were converted into double-stranded cDNA as previously described^14^. Short cDNA library was created by fragmentation using a Branson sonicator. The gel-purified double-strand fragments (DCF) were ligated immediately after the translation initiation code “ATG” of kanamycin. After transformation, only those E. coli colonies that carry the “in frame ATG-DCF-Kan+” were survived in the kanamycin LB plate. The “in-frame” DCFs were digested by BamH1/EcoRV and were re-ligated to the C-terminus of IFN in a lentiviral vector constructed in the lab^13^,^15^.

After packaging, lentiviruses were used to transfect 293T cells that carry the ISRE-GFP-Puro-Reporter system (Promega, WI). In this reporter system, “IFN-enhancer peptides” (IEP) will activate the ISRE promoter activity, leading to high expression of the copGFP/Puro reporter gene. The puromycin-resistant cells were collected and sorted for copGFP fluorescence by FACS Sorter (BD LSR Fortessa). The ISRE-responding IEP cDNAs were recovered by PCR and cloned into a pJet vector (Thermo Fisher, MA) for sequencing. Using this approach, we identified short cDNA fragments (IEP) that enhance the anti-tumor activity of IFN. Among the IEPs identified (Guo *et al.* unpublished data), three shared a consensus stretch of positively charged amino acids (Figs. S1A-S1B). Homologue search identified their origin from the C-terminus of placental growth factor-2 (PLGF-2) protein (Fig. S1C). Thus, we focused on the role of this short consensus IEP in this study (Fig. 1B).

### Recombinant plasmids and lentivirus production

The wild type IFNα expression construct was generated by amplifying full-length human IFNα cDNA from a mixed cDNA derived from human spleen and leucocytes using primers JH2240 (forward): 5′-TAGAAGAT**TCTAGA**GCCGCCACCATGGCCTTGACCTTTGCTTTACTG-3′ and JH2244 (reverse): 5′-ATTCGTCGAC**GATATC**TTATCATTCCTTACTTCTTAAACTTTCTTG-3′. The IFNα PCR product was digested with Xba1/EcoRV and cloned into a lentiviral vector containing copGFP and puromycin constructed in our lab^16^.

The synthetic interferon construct was generated by fusing the PLGF-2 IEP (Fig. 1B) in frame to the C-terminus of the human IFNα cDNA using overlapping PCR. The PLGF-2 IEP was amplified with primers JH2242 (forward): 5′-AGAAGTAAGGAAAGGAGGAGACCCAAGGGCAGGGGGA-3′ and JH2243 (reverse): 5′-TTCGTCGAC**GATATC**TCACAGGTGGCAGTCTGTGGGTCTC-3′, and was ligated to the C-terminus of IFNα cDNA by PCR ligation. To distinguish it from the parental IFNα, we named it as SIFα (synthetic IFNα). The SIFα PCR product was cloned into the same lentivirus vector using Xba1/EcoRV restriction sites. The putative structure of SIFα was predicted using the software as described^17^,^18^ (website: http://zhanglab.ccmb.med.umich.edu).

The same lentivirus vector without the interferon insert (vector) and the vector containing the IEP alone (IEP) were used as the control groups. All constructs were confirmed by DNA sequencing. Lentivirus vectors contained a green fluorescent protein (copGFP) gene to track lentiviral infection and a puromycin-resistant gene Puro + for stable cell selection.

For lentivirus packaging, constructed plasmids were co-transfected with pSPAX2 and pMD2G packing vectors using lipofectamine 2000 (Invitrogen, CA). The viral supernatants were collected 48 hrs and 72 hrs after transfection and used for cell transfection as previously described^14^,^19^.

### Infection of recombinant lentivirus

CFPAC1 and ASPC cells were plated at a density of 2 × 10^5^ cells in a six-well plate for 24 hours before transfection. Cells were infected with viruses using polybrene (10 mg/ml, Sigma, MO). After 24 hrs incubation at 37 °C, the medium was replaced with a fresh medium in the absence of polybrene. For stable transfection, cells were selected using puromycin (1 mg/ml, Invitrogen, CA) for 72 hrs.

### Enzyme-linked Immunosorbent Assay (ELISA)

Secretion of interferon in lentivirus-transfected pancreatic cancer cells was measured by ELISA. Pancreatic cancer cells were seeded into 6-well plate at a density of 2 × 10^5^ cells/well for 24 hrs and infected with lentiviruses. The culture supernatants were collected at different time points. Interferon concentration was determined by ELISA according to the manufacture’s instruction (PBL, NJ), and analyzed at an absorbance of 450 nm.

### Cell proliferation by sulforhodamine B Assay

The sulforhodamine B (SRB)-assay was performed to evaluate cell proliferation. Cells were seeded in 96-well plates at a density of 2 × 10^3^ cells/well for 24 hrs and infected with lentiviral supplements. Seven days after viral transduction, cells were fixed with trichloroacetic acid (1 h at 4 °C) and stained using SRB solution (0.4% SRB in 1% acetic acid). The optical density was measured at 510 nm after reconstitution of the dye in 150 μl 10 mM Tris buffer. The values were normalized to cell density of normal group cells (100%) and were corrected for the optical density values of PBS-treated cell. All experiments were performed in triplicate.

### Treatment of pancreatic cancer cells with the secreted IFNs

In this proof-of-concept study, we examined the role of the secreted SIFα and IFNα proteins in pancreatic cancer cells. For this, we expressed SIFα and IFNα in 293T cells. The supernatants (14 ml) containing the secreted interferons were collected and centrifuged at 4000 rpm for 10 min to get rid of cell debris. The supernatants were then passed through Amicon Ultra-15 centrifugal filter Unit (MW cut off 30 KD, EMD Millipore, CA) to get rid of large molecules. The flow-through containing the interferons (24.4 KD and 21.6 KD, respectively) was loaded onto Amicon Ultra-15 centrifugal filter Unit (MW cut off 10 KD, EMD Millipore, CA) to remove proteins with small molecule size. Interferons were measured with ELISA kit (PBL, CA). Based on the ELISA data, supernatants that contain the equal amount of secreted interferons were used for inhibition in cell growth.

### Western blot analysis

As previously described^20^, Western blot was used to detect the secreted SIFα and IFNα proteins in cell supernatants. Briefly, 293T cells were transfected with PBS, IFNα, SIFα, IEP and vector DNAs. Forty-eight hours after transfection, cell supernatants (20 μl) were separated on Mini-PROTEIN TGX gradient gel (Bio-Rad, CA) and transferred to a nitrocellulose membrane. After blocking with Odyssey Blocking buffer for 1 h, membranes were incubated with specific primary antibodies against IFNα (PBL, NJ) overnight at 4 °C. After washing with PBS for 4 times, the IRDye 680 secondary antibody (LI-COR, Lincoln, NE) was added and the infrared fluorescence was visualized with the Odyssey infrared imaging system (LI-COR, Lincoln, NE).

### Cell cycle analysis

Pancreatic cancer cells were harvested, washed with cold PBS, and processed for cell cycle analysis using flow cytometry. Briefly, cells were fixed in 75% ethanol and stored at −20 °C for later analysis. The fixed cells were centrifuged at 800 × rpm and washed with cold PBS twice. RNase A (20 μg/ml final concentration) and propidium iodide staining solution (50 μg/ml final concentration) were added to cells and incubated for 30 min at 37 °C in the dark. One hundred thousand cells were analyzed using a FACSCalibur instrument (BD Biosciences, CA) equipped with CellQuest 3.3 software. ModFit LT 3.1 trial cell cycle analysis software was used to determine the percentage of cells in different phases of cell cycle.

### Treatment with chemotherapeutic drug gemcitabin

Chemotherapeutic drug gemcitabin (GEM, Sigma, MO) was dissolved in phosphate-buffered salt solution (1 μM). For drug treatment, pancreatic cancer cells (CFPAC1, ASPC) were seeded into 12-well plate at a density of 1 × 10^5^ cells/well. After 24 hrs, GEM was added in the medium at desired concentrations. The medium was changed twice a week. After 1 week of GEM treatment, cell numbers were determined using the SRB assay. To better examine the therapeutic role of SIFα, we chose the dose of GEM that inhibited tumor cells about 10–20%. All experiments were repeated three times.

### Gene expression by RT-PCR

Total RNA was extracted from cultured cells using the Trizol reagent (Invitrogen, CA) following the manufacturer’s instructions. After removing the residual genomic DNA with DNase I (Sigma, MO), M-MLV Reverse Transcriptase (Invitrogen, CA) was used to synthesize cDNA^20^,^21^. For PCR, cDNA samples were amplified using Thermal Cycler (BIO-RAD). The mRNA expression level of ADPR, MX1, IFIT1, OAS2, P21, and CASPAS3 was quantified by normalizing over β-actin (housekeeping gene) as previously described^19^,^22^. The primers used for RT-PCR include: 1). ADPR-V1, JH2557 (forward): 5′-CAATGAATCCGCGGCAGGGGT-3′ and JH2558 (reverse): 5′- TCTGGGATCTGCCCCTTGAG-3′; 2). MX1, JH2769 (forward): 5′- CASTATGAGGAGAAGGTGCG-3′ and JH2770 (reverse): 5′- TCAGCACCAGMGGGCATCTGGT-3′; 3). IFIT1, JH2767 (forward): 5′-GCTGCCWMMTTTACAGCAACCATG-3′ and JH2768 (reverse): 5′-CAGGCAMAGTTGCCCCAGGTC-3′; 4). OAS2, JH2771 (forward): 5′-GWKGGTWTATCCAGGAAWACCT-3′ and JH2772 (reverse): 5′-GRACAAGGGTACCATCGGAG-3′; 5). P21, JH839 (forward): 5′-GTGGACCTGTCACTGTCTTGTAC-3′ and JH840 (reverse): 5′-GCTTCCTCTTGGAGAAGATCAGC-3′; 6). Caspase3, JH2629 (forward): 5′-ATGGAGAACACTGAAAACTCAG-3′ and JH2630 (reverse): 5′-CAGACCGAGATGTCATTCCAG-3′.

### Cell binding assay of SIFα

The binding of the interferons to the cell membrane receptor was compared by FACS following the method as previously described^23^,^24^. Pancreatic cancer cells were collected and stained with Trypan blue to make sure that viable sells were >90%. Cells (1 × 10^6^ cells/tube in PBS) were incubated with equal amount of the secreted SIFα and IFNα for 1 h at 37 °C. After wash with PBS, cells were incubated with FITC-conjugated IFNα antibody (PBL, NJ), according to the manufacture’s instruction. The FITC-conjugated mouse IgG (Abcam, MA) was used as the isotype control. Cells were analyzed using FACSCalibur (BD Biosciences, CA) equipped with CellQuest 3.3 software to calculate the Median Fluorescence intensity (MFI).

### Statistical analysis

All experiments were performed in triplicate, and the data were expressed as mean ± SD. The comparative *C*T method was applied in the quantitative real-time RT-PCR assay according to the delta-delta *C*T method^13^,^25^. Data were analyzed using SPSS software (version 20.0; SPSS, IL). One-way *ANOVA (Bonferroni test)* was used *to* compare statistical differences for variables among treatment groups. Results were considered statistically significant at P ≤ 0.05.

## Results

### Synthetic IFNα-IEP construct

To potentiate the therapeutic activity of interferon alpha, we used a “cDNA in-frame fragment” screening approach to identify short peptides that enhance the activity of interferons (Fig. 1A). Among the identified IFN enhancer peptides (IEP), three contained a consensus amino acid sequence consisting of poly-arginine/lysine positively charged residues. By homologue search, we found that this consensus IEP was related to the C-terminus of placental growth factor-2 (PLGF-2) protein (Fig. S1). Interestingly, a peptide containing this IEP is known to enhance the function of vascular endothelial growth factor-A, platelet-derived growth factor-BB, and bone morphogenetic protein-2^11^.

To determine if this PLGF-derived positively charged peptide is able to potentiate the activity of IFNα, we constructed a synthetic interferon alpha protein (SIFα) by fusing this positively charged IEP peptide to the C-terminus of IFNα (Fig. 1B). The synthetic gene was placed under the control of a viral CMV promoter in the lentiviral expression vector. For comparison, the wild type IFNα was cloned into the same lentiviral vector. The lentiviral vector without the insert was used as the negative control. The predicted protein structure of SIFα was shown in Fig. 1C.

With the fluorescence of copGFP in the viral vector, we found that two pancreatic cancer cell lines (ASPC and CFPAC1) were efficiently transduced by the synthetic interferon virus (Figs. S2A, S2C). After lentiviral infection, we collected cell supernatants and used ELISA to quantitate the secreted interferon. Both pancreatic cancer cells secreted IFNα in the cell supernatant, with the peak at 48 hrs after viral transfection (Figs. S2B, S2D). Addition of the IEP peptide did not significantly affect the secretion of IFN in these two tumor cells.

### Synthetic SIFα inhibits cell growth in pancreatic cancer cells

**Figure 2:**
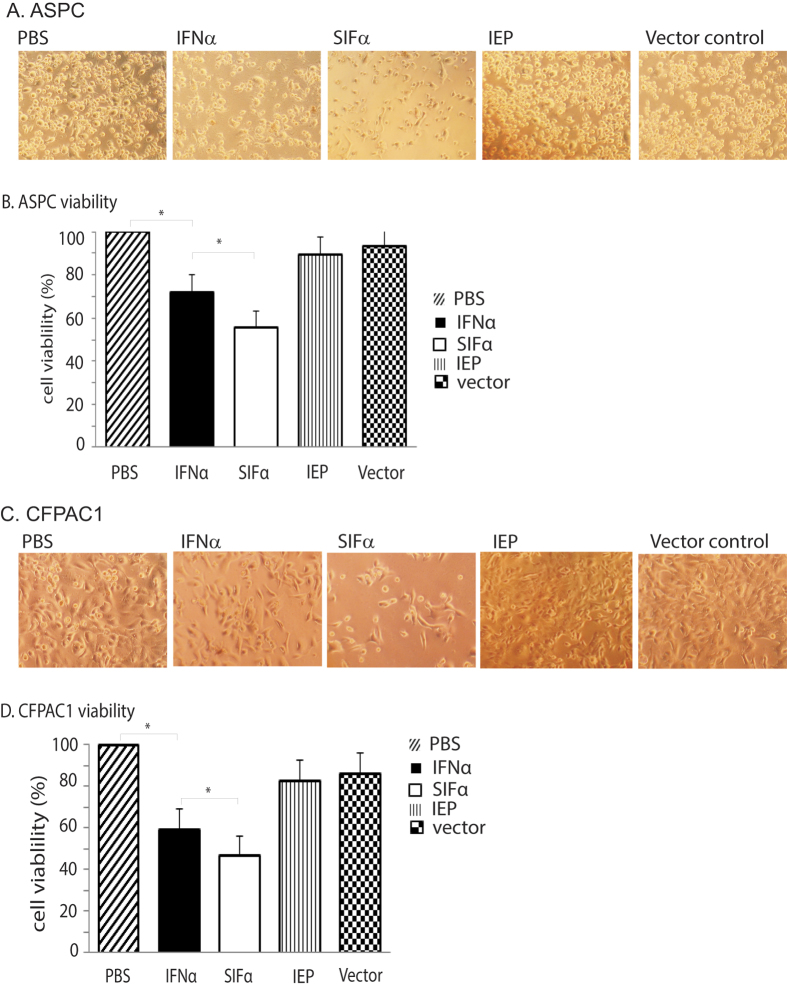
Synthetic interferon SIFα inhibits tumor cell growth. (**A**) Inhibition of cell growth in ASPC pancreatic cells. ASPC cells were transfected with lentiviruses carrying IFNα, SIFα, IEP, and vector control. PBS was used as a negative control. Photos were taken on day 7 after viral transduction. (**B**) Cell viability in ASPC pancreatic cells. Cells were collected on day 7 following lentiviral transfection. All data shown are mean ± SD from three independent. *p < 0.05 as compared with controls and with the wild type IFNα group. (**C**) Inhibition of cell growth in CFPAC1 pancreatic cells. (**D**) Cell viability in CFPAC1 pancreatic cells. All data shown are mean ± SD from three independent. *p < 0.05 as compared with controls and with the wild type IFNα group.

We examined the role of the synthetic SIFα on cell proliferation in pancreatic cells. Pancreatic tumor cells were transfected with lentiviruses carrying the wild type IFNα, the synthetic SIFα, and the empty vector, respectively. As expected, the wild type IFNα inhibited the growth in ASPC cells compared with that in the PBS control (Fig. 2A, panels 2 vs 1). However, SIFα showed the best inhibition among the treated groups (Fig. 2A, panel 3). The PLGF-derived positively charged peptide (IEP) alone did not interfere with the cell growth (Fig. 2A, panel 4).

We then compared the activity between the wild type IFNα and the synthetic SIFα by the sulforhodamine B (SRB)-assay. Again, we found that the synthetic SIFα exhibited a significantly greater inhibition of tumor cell growth than did the wild type IFNα in ASPC pancreatic cancer cells (Fig. 2B, p < 0.05).

We also tested the activity of SIFα in a second pancreatic cancer cell line CFPAC1. Similarly, we found that the synthetic SIFα showed a significantly higher activity in inhibiting CFPAC1 cancer cell growth (Fig. 2C,D, p < 0.05).

### Inhibition of tumor cell growth by the secreted interferons

**Figure 3:**
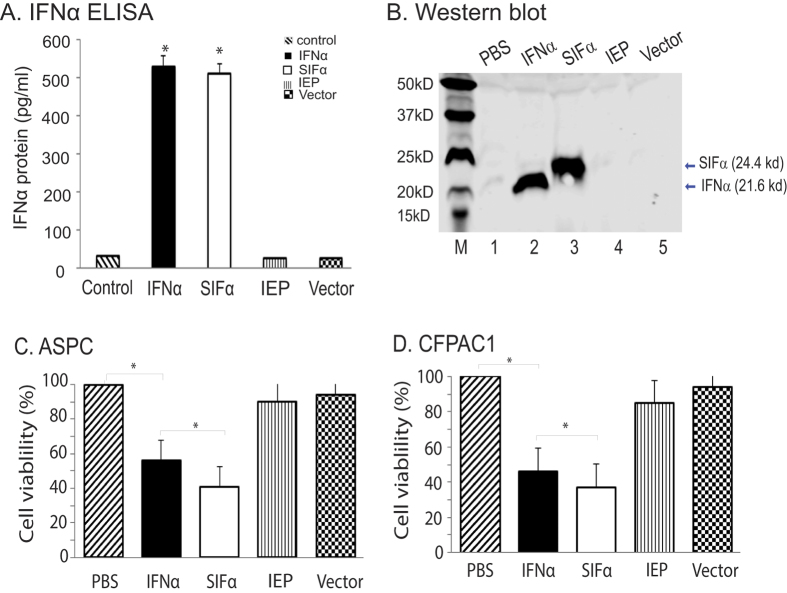
Inhibition of tumor cells by the secreted recombinant interferons. (**A**) Quantitation of the secreted IFN in cell supernatants. 293T cells were transfected with SIFα, IFNα, IEP, and vector plasmid DNAs using Lipofectamin 2000. Forty-eight hours after transfection, the cell medium was collected for ELISA quantitation of the secreted IFN. *p < 0.05 as compared with the PBS and IEP controls. (**B**) Western blot of SIFα. Supernatants (20 μl) collected from the IFNα, SIFα, IEP, and vector-transfected 293T cells (**A**) were separated on Mini-PROTEIN TGX gradient gel (Bio-Rad, CA), transferred to a nitrocellulose membrane, probed with IFNα antibody, and detected by the Odyssey infrared imaging system. SIFα: 24.4 kD, IFNα: 21.6 kD as estimated by ExPASy (http://web.expasy.org/cgi-bin/compute_pi/pi_tool). (**C–D**) Inhibition of cell growth of ASPC (**B**) and CFPAC1 (**C**) cancer cells using cell supernatants that contain the equal amount of the secreted interferons. Cell growth was measured by sulforhodamine B (SRB)-assay. All data shown are mean ± SD from three independent. *p < 0.05 as compared with the PBS control and with the wild type IFNα group.

After confirming the therapeutic effect of IFNα in the form of lentivirus, we then examined if the secreted interferons in the supernatants had the same antitumor activity. For this, we transduced 293T cells with lentiviruses and collected cell supernatants that contain the secreted interferons. ELISA assay was used to measure the secretion of IFN in the medium (Fig. 3A). Western blot confirmed the production of the full length of SIFα protein (Fig. 3B). Two pancreatic cells were treated with cell supernatants containing the equal amount of IFN. Cell proliferation was measured with the sulforhodamine B (SRB). As the control, the supernatant from the empty vector virus-infected 293T cells did not affect cell growth. Supernatants from both the wild type IFNα and the synthetic SIFα virus-infected 293T cells inhibited cell growth, with the synthetic SIFα showing the best antitumor activity in both pancreatic cancer cells (Fig. 3C).

### SIFα potentiates the antitumor therapy by low dose of gemcitabin

**Figure 4:**
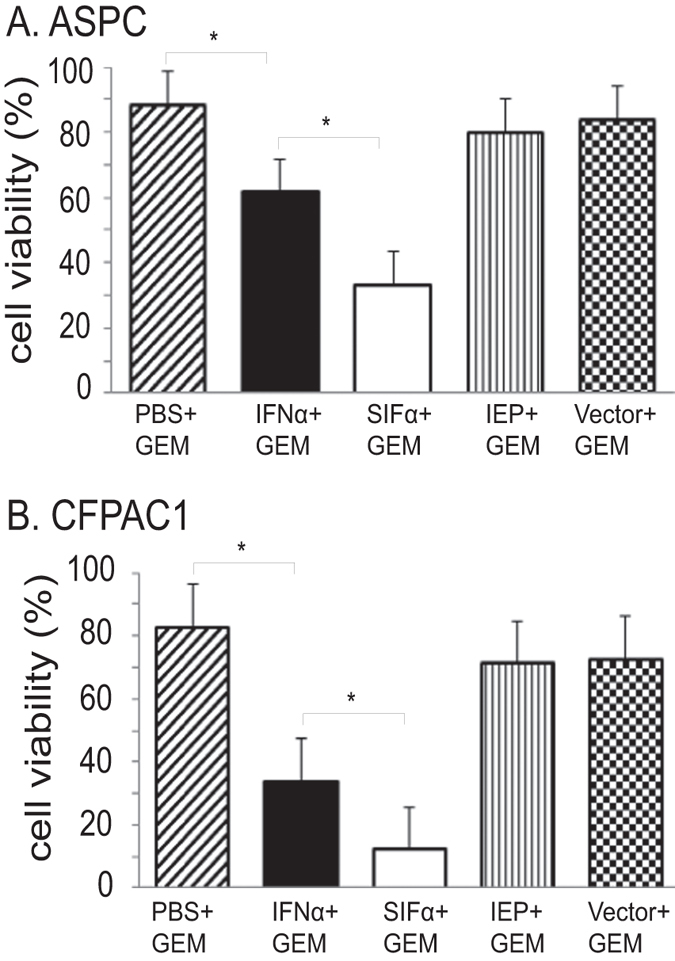
SIFα potentiates the therapeutic effect of Gemcitabin (GEM). Pancreatic cancer cells ASPC (**A**) and CFPAC1 (**B**) were transfected with interferon lentiviruses in the presence of the low dose of GEM. Cell growth was measured by sulforhodamine B (SRB)-assay. All data shown are mean ± SD from three independent. *p < 0.05 as compared with controls and with the wild type IFNα group.

Gemcitabin (GEM) has been used as the first line chemotherapeutic agent for the treatment of pancreatic cancer. It causes DNA damage in cancer cells at the “S” phase of the cell cycle. We then tested if the engineered SIFα was able to potentiate the activity of this chemotherapeutic drug in pancreatic cancer cells. For this, a low dose of GEM was used to induce about 10–20% inhibition of cell growth in pancreatic cells. In both pancreatic cancer cell lines, we found that the synthetic SIFα showed a significantly higher activity than did wild type IFNα in potentiating the antitumor cell growth mediated by a low dose of GEM (Fig. 4, p < 0.01).

### Synthetic SIFα induces the S-G2/M blockage

**Figure 5:**
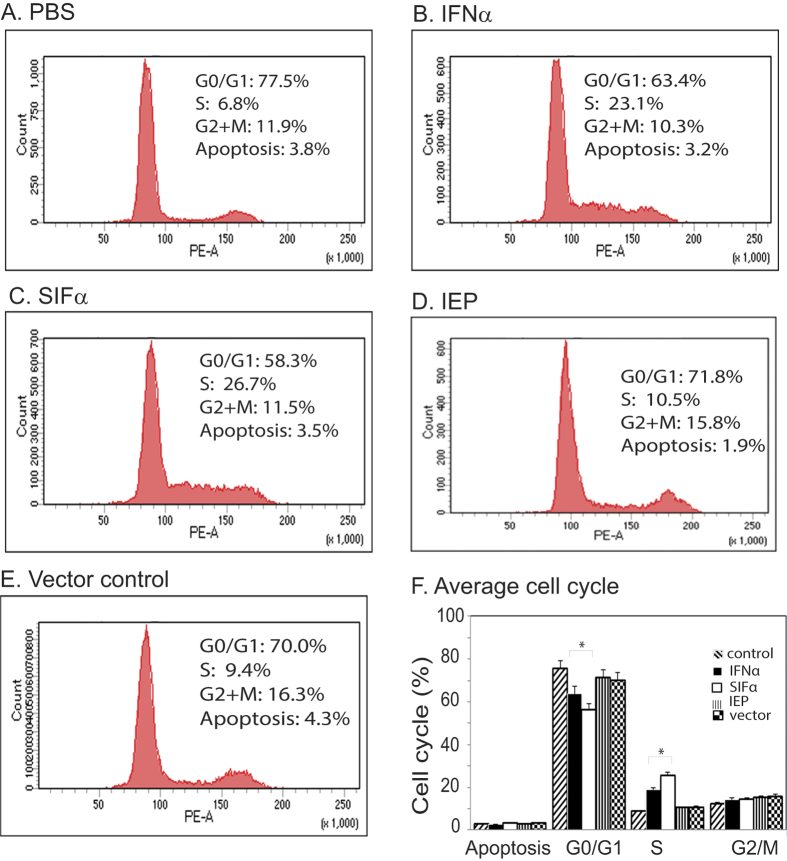
SIFα induces S-G2/M phase blockage. CFPAC1 tumor cells were transfected with interferon lentiviruses and submitted to cell cycle analysis (**A–E**). Three independent cell cycle analyses were performed and averaged for each treatment group (**F**). *p < 0.05 as compared with the wild type IFNα group.

We then examined the effect of the SIFα treatment on cell cycle. After the treatment with SIFα, cells were collected and subjected to cell cycle analysis. We found that the synthetic interferon inhibited cell growth by blocking cells at the S-G2/M transition phase (Fig. 5).

### Activation of the STAT1 pathway by the engineered IFNα

**Figure 6:**
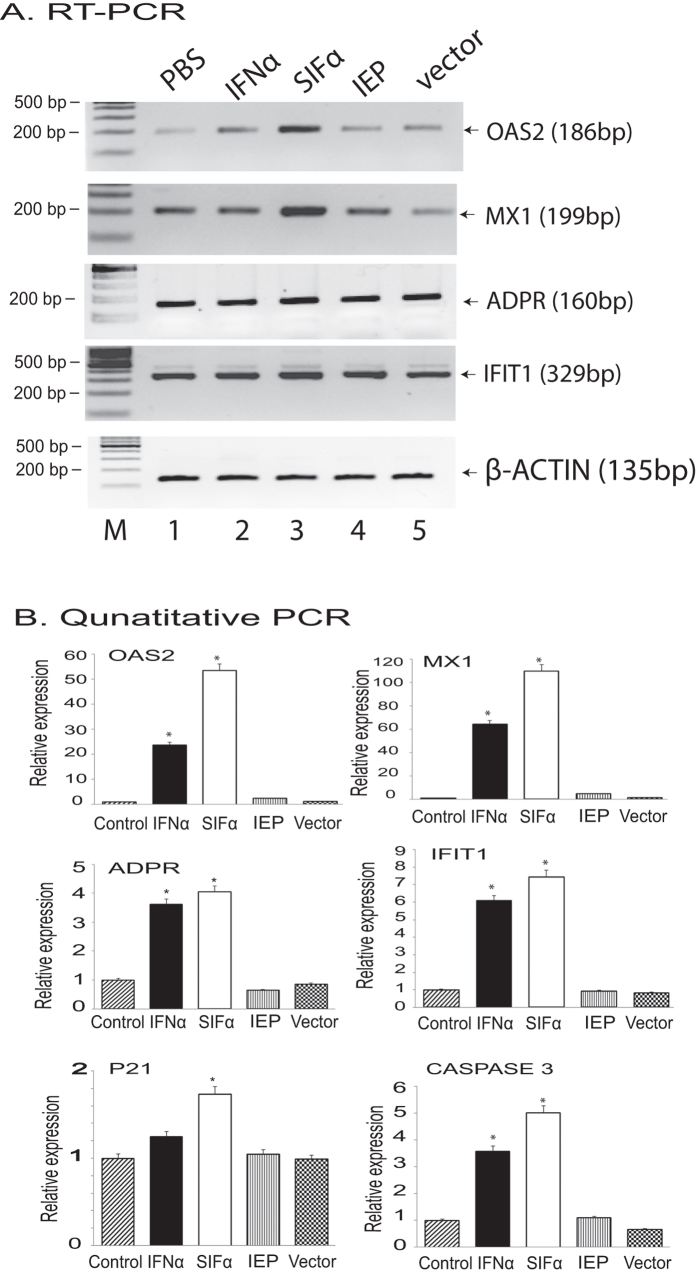
SIFα activates the STAT1 signal pathway. (**A**) RT-PCR of the STAT1 signal pathway genes in ASPC tumor cells. β-ACTIN was used as the internal control for PCR reaction. (All the agarose gels had been run under the same experimental conditions. The original full-length gels are included in the supplementary information). (**B**) Quantitative analyses of the activation of the STAT1 signal pathway and the apoptosis pathway. All data shown are mean ± SD from three independent. *p < 0.05 as compared with the PBS control and with the wild type IFNα group.

To examine the potential mechanism underlying this enhanced antitumor activity by SIFα, we used RT-PCR to quantitate the expression of interferon pathway genes. Transfection with lentiviruses carrying either the empty vector or the PLGF-derived positively charged peptide (IEP) alone did not significantly affect the expression of the STAT1 pathway genes, including OAS2, MX1, ADPR, and IFIT1 (Fig. 6A, lanes 4–5). However, SIFα treatments upregulated the transcription of these pathway genes (Fig. 6A, lane 3) in parallel with the growth inhibition of pancreatic cancer cells. Quantitative PCR also confirmed the significant upregulation of these STAT1 pathway genes by SIFα (Fig. 6B). In addition, SIFα also significantly activated two genes involved in the apoptosis pathway (P21, CASPASE 3).

### IEP enhances the IFN binding to the cell membrane

**Figure 7:**
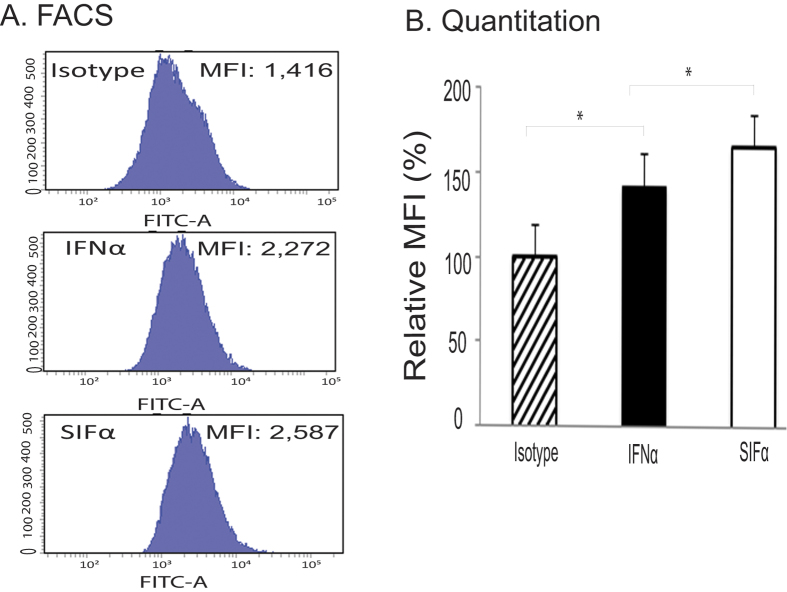
SIFα enhanced cell binding of IFN in CFPAC1 cancer cells. (**A**) Cell binding assayed by FACS. After ELISA quantitation, the cell supernatants containing equal amount of the secreted interferons (SIFα and IFNα) were incubated with CFPAC1 cancer cells. The FITC conjugated-IFNα antibody was used for FACS quantitation of the ability of cell binding. The FITC mouse IgG was used as the isotype control. The Median Fluorescence Intensity (MFI) was calculated to evaluate the cell binding. (**B**) Relative MFI in SIFα- and IFNα-treated cells. MFI was standardized as the percentage of the isotype control group. Data represent mean ± SD of three independent repeats. * Significantly higher binding to the pancreatic cancer cells (p < 0.05).

The effects of IFNα are mediated through the binding and interaction with the specific cell surface type I IFN receptor. The IEP peptide selected in this study contains a short stretch of positively charged amino acids. We thus explored if IEP functions through a mechanism by promoting the binding of IFN to the cell membrane. For this, we used cell binding assay to compare the binding ability of the soluble SIFα and IFNα in pancreatic cancer cells. The Median Fluorescence intensity (MFI) was calculated to assess the binding activity. As seen in Fig. 7, SIFα showed a significantly higher binding ability to the IFNα receptor on the pancreatic cell membrane than did IFNα (p < 0.05).

## Discussion

Systemic and immune therapies show clinical benefit in extending patient’s survival. Interferon alpha (IFNα), a natural potent pleiotropic cytokine, has been used to treat a variety of malignancies^26^. IFNα is produced predominantly by natural killer (NK) and natural killer T (NKT) cells as part of the innate immune response, and by CD4 Th1 and CD8 cytotoxic T lymphocyte (CTL) effector T cells in antigen-specific immunity^27^. Binding of this cytokine to its surface receptor initiates a cascade of events that induce the phosphorylation of JAK1 and TYK2 kinases, followed by the activation of the signal transducer and activation of transcription (STAT) family transcription factors^28^,^29^,^30^,^31^. The activated STAT complex is subsequently translocated to the nucleus, where it induces transcription of a number of genes related to cell-cycle arrest and apoptosis, resulting in both apoptotic and nonapoptotic cell death^29^,^30^,^32^ as the molecular basis for antiviral and antitumor therapy.

Although IFNα is a natural cellular cytokine against viral infection and tumors, its clinical application as tumor therapy has been greatly restricted due to its toxicity and relatively low biological activity. Using a “cDNA in-frame fragment library” screening assay, we identified three short “IFN enhancer peptides” (IEPs) that contain a consensus stretch of positively charged amino acids from placental growth factor-2 (PLGF-2)(Fig. 1). When fused to the C-terminus of IFNα, this short peptide significantly potentiates the antitumor activity of IFNα in pancreatic cells. As compared with the wild-type IFNα, the synthetic Interferon alpha (SIFα) exhibited a significantly higher biological activity in inhibiting tumor cell growth. In a parallel study, we also demonstrate that when fused to the C-terminus of interferon gamma, this short positively charged peptide significantly potentiates the antitumor activity of interferon gamma (IFNγ) in glioblastoma U87 cells (Liu *et al.* unpublished data). The synthetic IFNγ protein inhibits tumor cell growth, invasion and tumor colony formation at a higher efficiency than the unmodified IFNγ.

Currently, it is not clear as how this short peptide enhances antitumor activity of IFNα. The PLGF-2 peptide is a positively charged peptide containing 11 arginine (K) and lysine (L) residues enriched at its N-terminus (Fig. 1B). Positively charged peptides have been used as cell permeable peptides (CPPs) to facilitate the ability of recombinant proteins to cross cell membranes^33^,^34^,^35^, including the HIV-1 TAT^36^, MPG^37^ and herpes simplex virus-type 1 virus VP22 proteins^38^. Attaching CPPs to a recombinant protein helps deliver the cargo into living mammalian cells by direct penetration and endocytosis^33^,^39^,^40^,^41^. It is worth noting that this short peptide itself does not have any effect on cell proliferation. Therefore, we believe that the potentiation of the IFNα activity by this PLGF-2 PCP peptide may be related to its positively charged amino acids. The activity of growth factors is orchestrated by their binding to the extracellular matrix (ECM)^42^. The ECM bound IFNα is more active than its soluble counterpart^43^. Martino *et al.* showed that PLGF-2 aa123–144 peptide was able to enhance the function of several growth factors, including vascular endothelial growth factor-A, platelet-derived growth factor-BB, and bone morphogenetic protein-2^11^. It is assumed that the activity of PLGF-2 aa123-144 peptide is related to its extraordinarily strong and promiscuous interaction with extracellular matrix (ECM) components, thus facilitating its localization and spatially regulating the signaling of the growth factors. Three IFNα enhancer peptides isolated from the cDNA in-frame library screening contain the PLGF-2 aa123-144. Thus, it is possible that they may use a similar mechanism of ECM binding to potentiate the activity mediated by IFNα. Through the binding of its negatively charged components in the extracellular matrix, the PLGF-2 peptide may facilitate SIFα’s access to its membrane receptor for the enhanced activity. Indeed, using the cell binding assay, we demonstrate that the IEP, when fused to the C-terminus of IFNα, promotes the binding of IFN to the cell membrane receptor (Fig. 7).

The molecular mechanisms underlying the antitumor effects of interferons have yet to be elucidated. A variety of cellular responses, including inhibition of cell growth and induction of apoptosis are induced by IFNs^44^,^45^,^46^. Apoptotic induction by this cytokine has been proposed to be of importance for both its anti-tumor/anti-viral responses. IFNs initiates apoptosis through the activation of both intrinsic and extrinsic pathways, or by the stress kinase cascade^47^,^48^,^49^. The production of type I IFN can be magnified by a positive feedback loop mechanism, probably through IRF8 that activates basic transcription machinery to the IFN promoters^50^,^51^,^52^. In this study, we found that addition of the short IEP peptide to the C-terminus of IFNα did not significantly affect the secretion of the cytokine in two pancreatic cancer cells (Fig. S2). Thus, it appears that SIFα may enhance the function of the interferon by an alternative mechanism. Our cell binding data suggest that IEP may play a role in promoting the binding of IFN to the cell membrane receptor (Fig. 7).

IFNs can affect mitotic cycle and induce cell cycle arrest mostly at the G1 phase or a prolongation and accumulation of cells in the S-phase, primarily due to the disability to complete DNA replication following the down-regulation and impaired activity of cyclin and cyclin-dependent kinases^53^,^54^,^55^,^56^. In this study, we examined the effect of synthetic SIFα on cell cycle. Interestingly, we found that SIFα inhibited cell growth primarily by blocking cells at the S phase (Fig. 5), thus making the pancreatic cancer cells more susceptible to the therapy of low dose of gemcitabine (Fig. 4).

It should be emphasized that this is a proof-of-concept study launched to examine the role of SIFα. For convenience, we simply used a lentivirus vector system to deliver SIFα to pancreatic cancer cells. It is not guaranteed if these data will be equally translated into preclinical and clinical studies, particularly using recombinant proteins. Thus, it would be more practical to confirm if SIFα, when delivered in the form of purified recombinant proteins, is able to potentiate the antitumor activity in an *in viv*o tumor study. In addition, unmet clinical needs of IFN treatment exist, including systemic toxicity and short half-life of molecule. Thus, pharmacokinetic and pharmacodynamic studies of the purified recombinant protein are needed to examine if the addition of the positively charged IEP will affect intracellular signaling such as protein synthesis and if the IEP will affect the structural stability of IFN.

In summary, we have identify a short positively charged peptide (IEP) using the “cDNA in-frame” fragment/IFN-responding ISRE-luciferase reporter system. When fused to the C-terminus, IEP significantly potentiates the antitumor activity of interferon alpha in pancreatic cancer cells. The synthetic SIFα inhibits tumor cell growth and blocks cells at the S-G2/M phase by activating the STAT1 pathway. In this proof-of-concept study, we only tested a single consensus IEP that contains a short stretch of positively charged amino acids. It is not clear if the originally identified three IEP peptides would have a better antitumor effect than the tested IEP. Ideally, these positively charged IEPs need to be further optimized using technologies, like phage display, to robust the antitumor activity.

## Additional Information

**How to cite this article**: Yin, H. *et al.* Antitumor potential of a synthetic interferon-alpha/PLGF-2 positive charge peptide hybrid molecule in pancreatic cancer cells. *Sci. Rep.* **5**, 16975; doi: 10.1038/srep16975 (2015).

## Supplementary Material

Supplementary Information
