# Supplementary material for: Antitumor potential of a synthetic interferon-alpha/PLGF-2 positive charge peptide hybrid molecule in pancreatic cancer cells
Source: Sci Rep. 2015 Nov 20;5:16975. doi: 10.1038/srep16975 (PMC4653758; doi:10.1038/srep16975)
Supplement: Supplementary Information [file srep16975-s1.doc]

**Antitumor potential of a synthetic interferon-alpha/PLGF-2 positive charge peptide hybrid molecule in pancreatic cancer cells**

Hongmei Yin1,2¶, Naifei Chen1,2¶, Rui Guo^1,2^, Hong Wang^1,2^, Wei Li^1^, Guanjun Wang^1^, Jiuwei

Cui1#, Haofan Jin1#, Ji-Fan Hu1,2#

1 Stem Cell and Cancer Center, First Hospital, Jilin University, Changchun, Jilin 130021, China

2 Stanford University Medical School, Palo Alto Veterans Institute for Research, Palo Alto, CA

94304, USA

¶ Equal contribution

# Correspondence to: Ji-Fan Hu, M.D., Ph.D., Palo Alto Veterans Institute for Research, Palo

Alto, CA 94304, USA, tel: +1-650-493-5000, x63175, fax: +1(650)-849-1213, E-mail:

[jifan@stanford.edu](mailto:jifan@stanford.edu); Haofan Jin, Cancer Center, First Hospital, Jilin University, Changchun, Jilin

130021, China, tel: +86-431-8878-2178, e-mail: [Kinhf1968@126.com](mailto:Kinhf1968@126.com); or Jiuwei Cui，Cancer

Center, First Hospital, Jilin University, 71 Xinmin Street, Changchun 130021, China, Tel: +86

431-8878-2178, e-mail: [cuijiuwei@vip.qq.com](mailto:cuijiuwei@vip.qq.com).

Supplementary Figure S1

A. IEP library screening

cDNA fragments

(DCF)

Kanamycin

in-frame ligation

IFNα-DCF ligation ISRE/copGFP

screening vector

IFNα-enhaner peptides (IEP)

IFNα-IEP fusion protein

Tumor cell testing

B. Three IEPs sharing positively charged AAs

IEP-1: IEP-2: IEP-3:


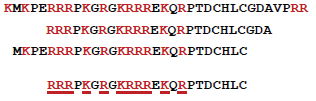


Consensus IEP:

C. Alignment to PLGF-2

Gene

E1 E2 E3 E4 E5 E6 E7

Exons

PLGF-2

IEP1

IEP2

IEP3

**Figure S1. Screening of interferon enhancer peptides (IEP).**

A. Schematic diagram of IEP library screening. Double-strand cDNAs (DCF) from fetal heart mesenchymal cell-derived fibroblast like cells are ligated in frame with translation initiation code “ATG” of kanamycin. The “in-frame” DCFs are selected by kanamycin and are fused to the C-terminus of IFN. Using the ISRE/copGFP/Puro+ reporter system, IEPs are identified and cloned for testing their antitumor activity.

B. Three identified IEPs that share a consensus stretch of positively charged amino acids (red).

C. Alignment of the three IEPs to the C-terminus of PLGF-2.

Supplementary Figure S2

A. Lentiviral infection in CFPAC1

B. IFNα secretion in CFPAC1

PBS


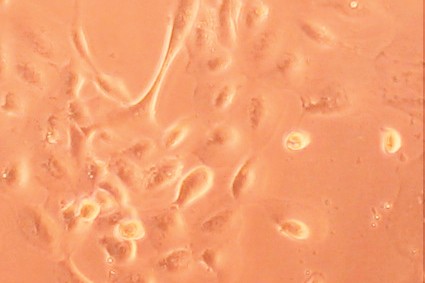


SIFα


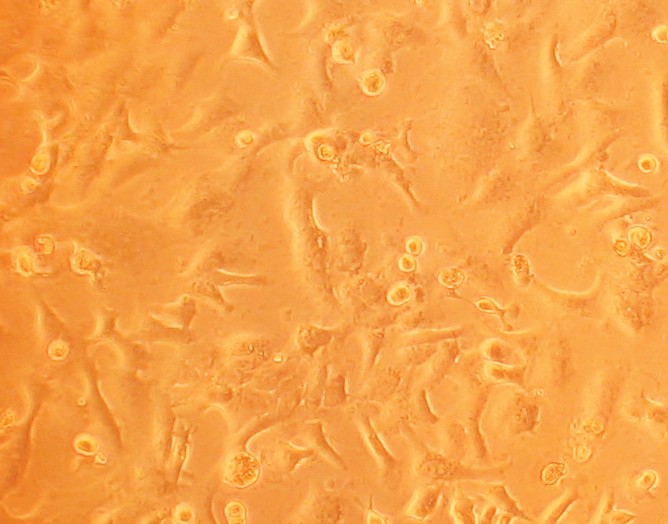


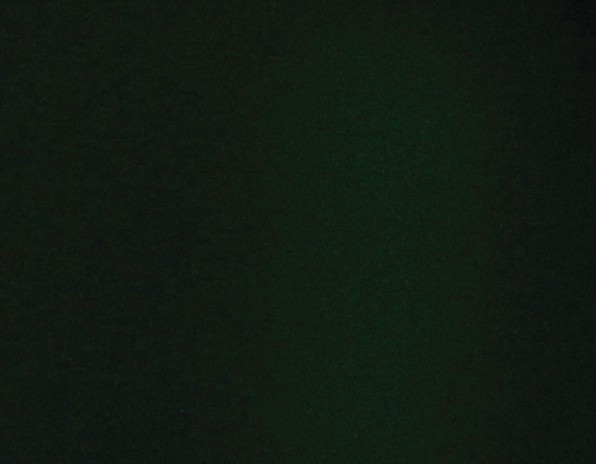
 IFNα


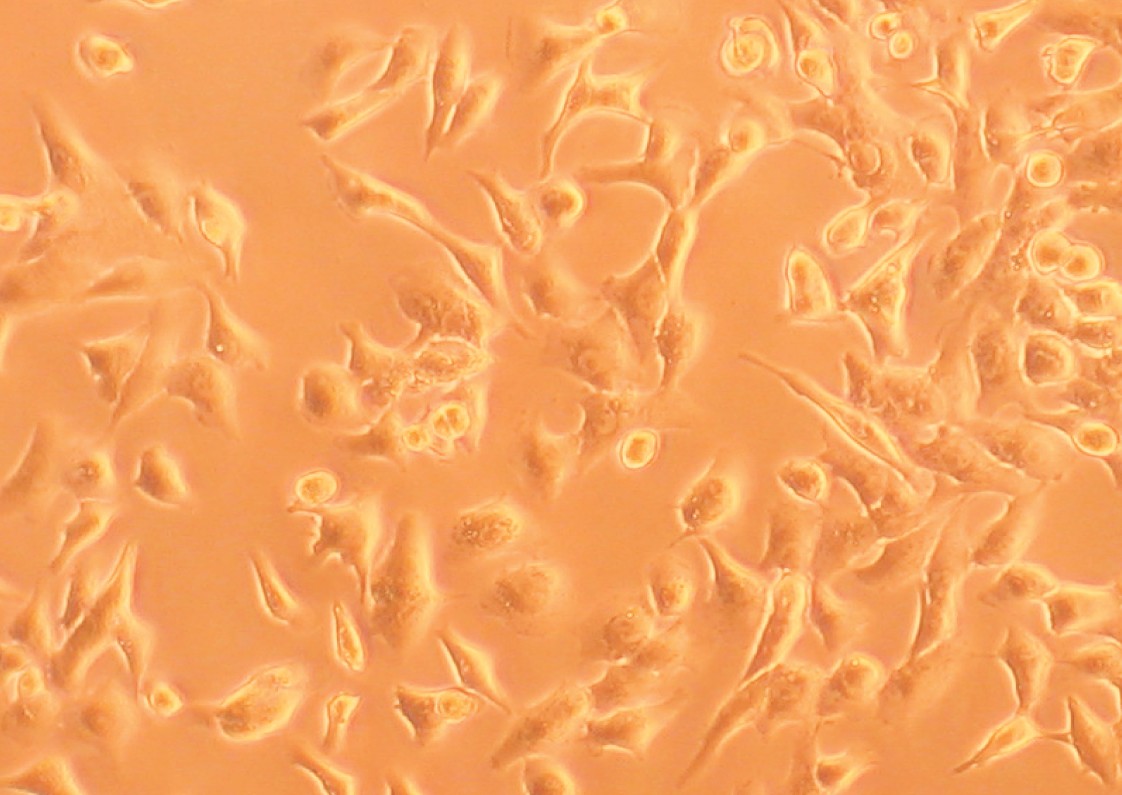


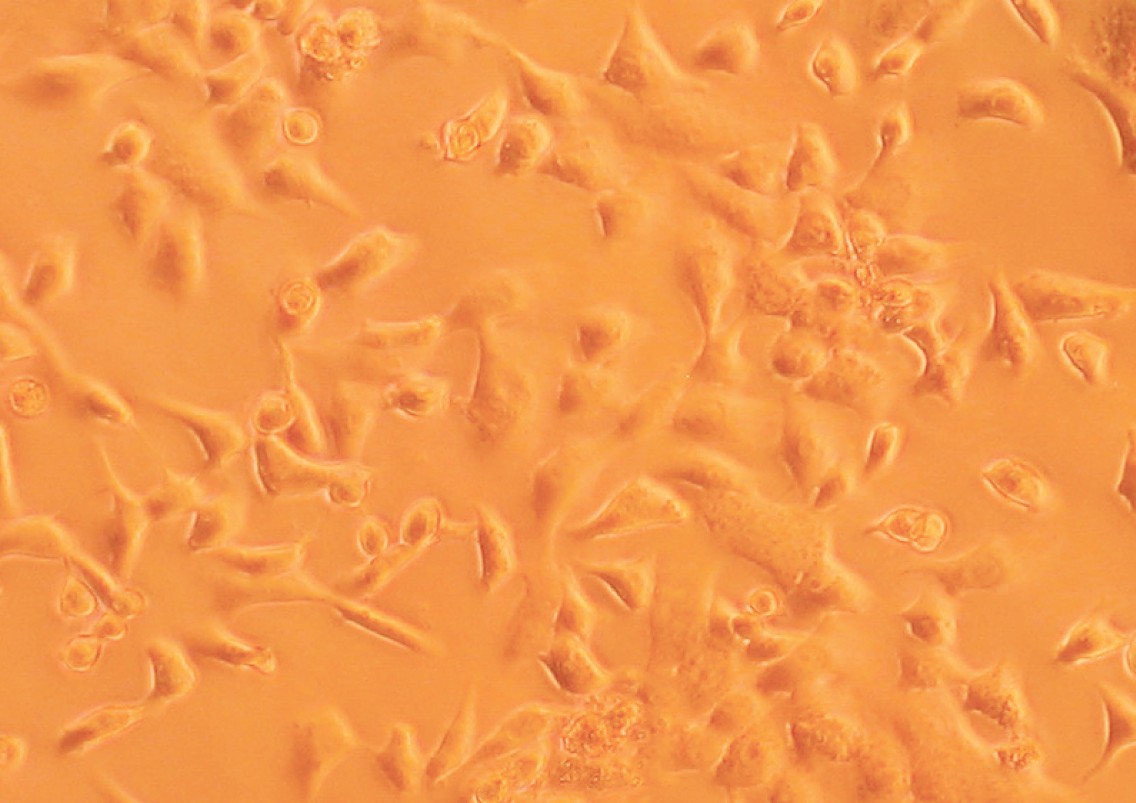


IEP


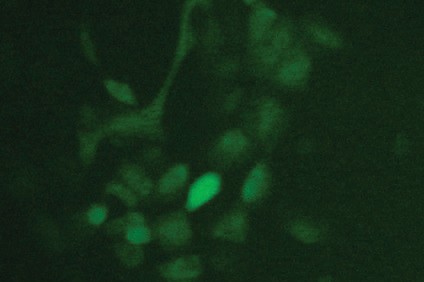


600


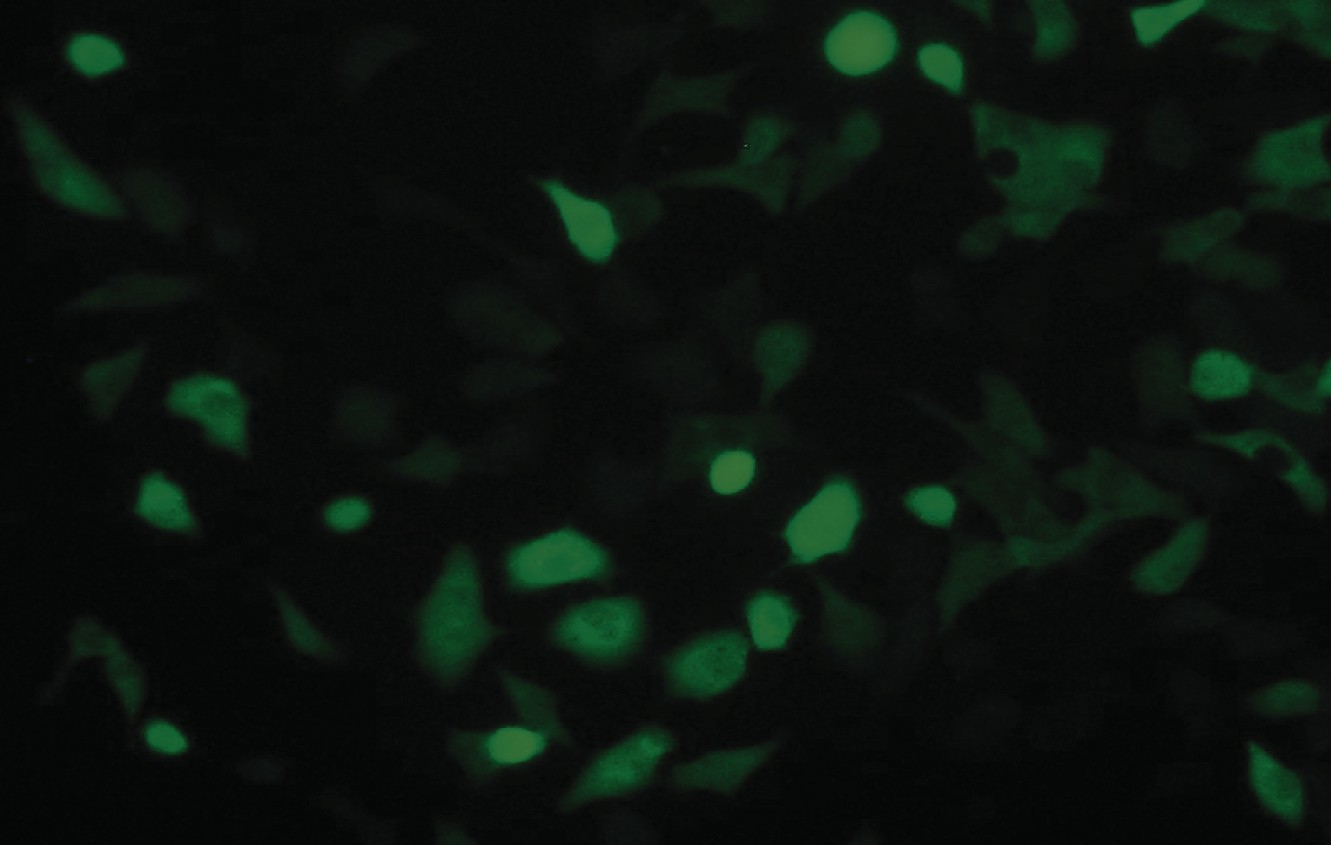


500

IFNα secretion (pg/ml)

400

300


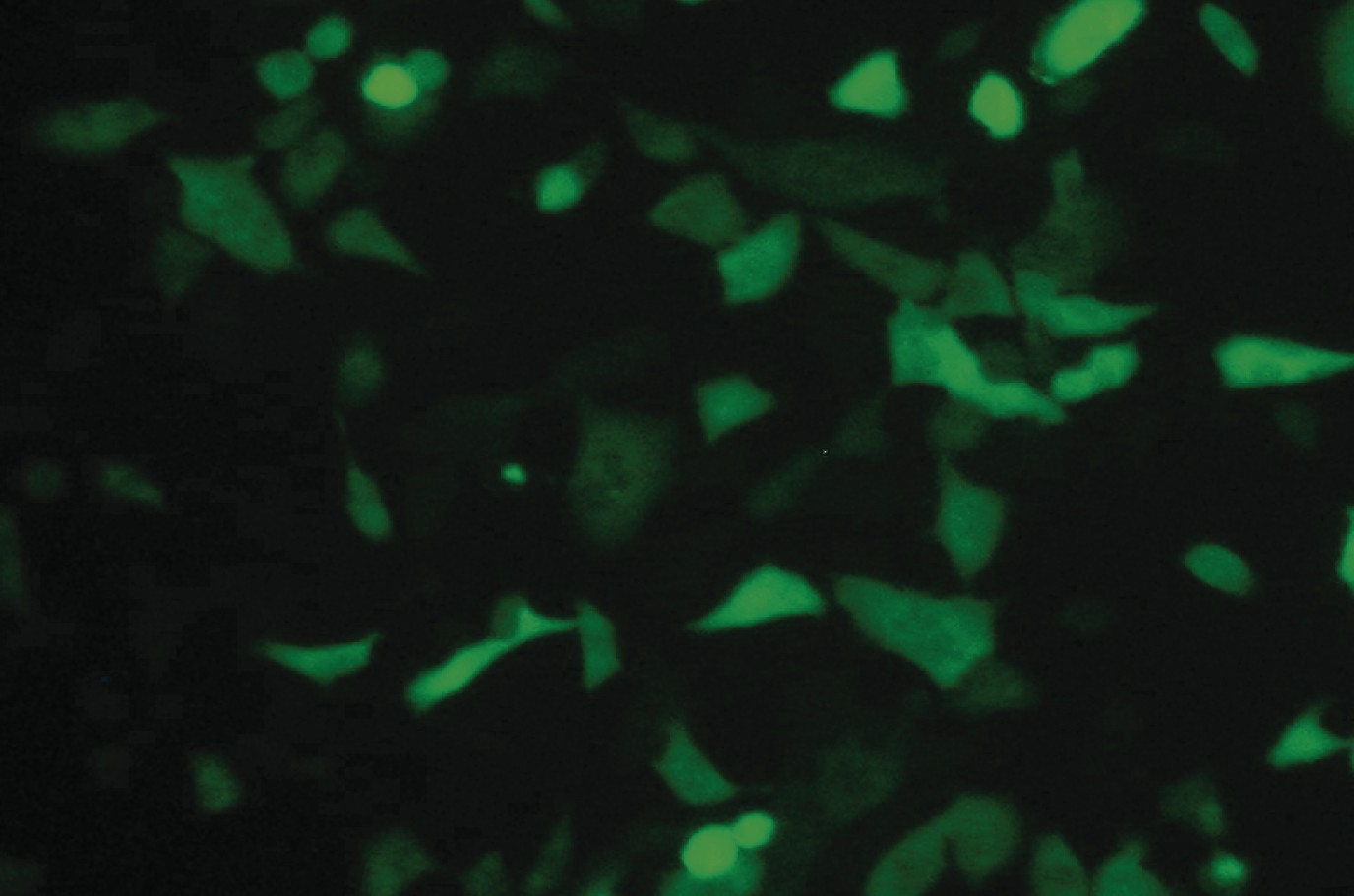


200

100


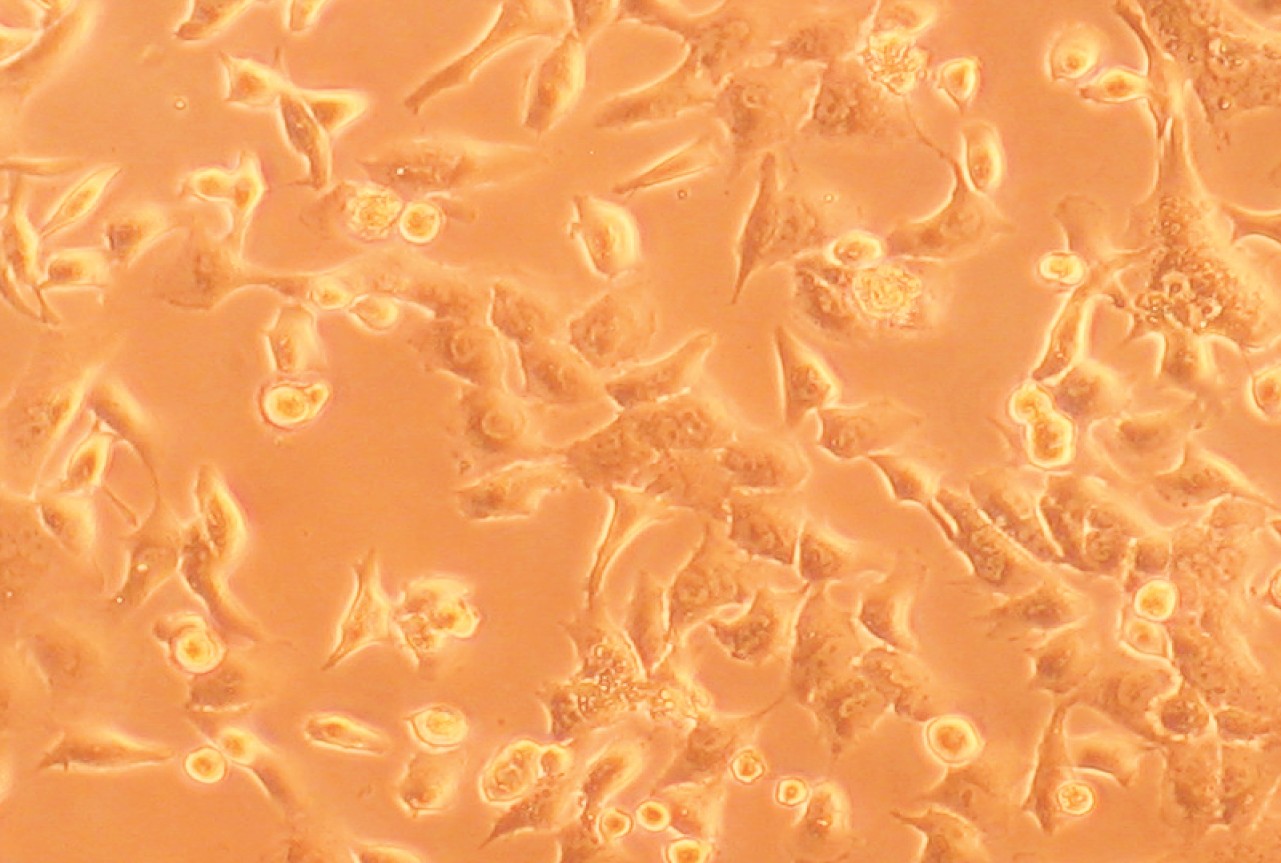


PBS * *

IFNα

SIFα

IEP *

vector *

* *

Vector


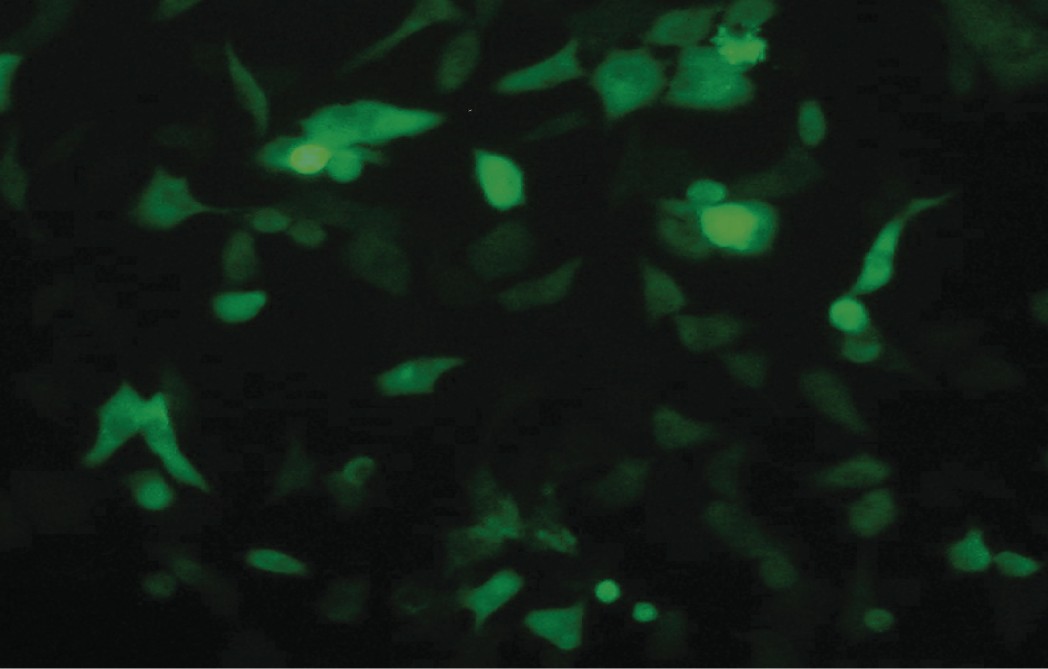


0

24 h 48 h 72 h

C. Lentiviral infection in ASPC D. IFNα secretion in ASPC

PBS


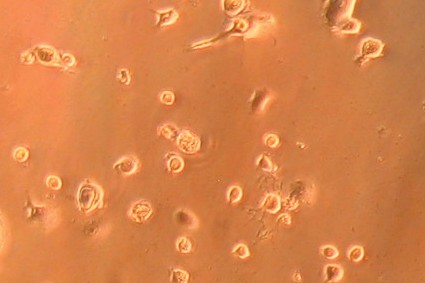


SIFα


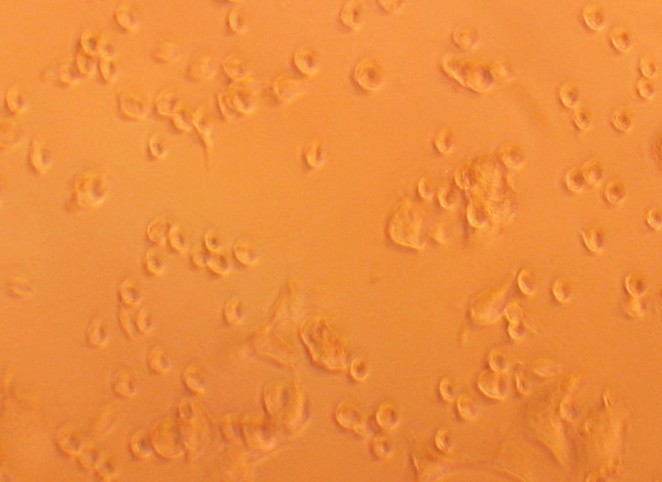


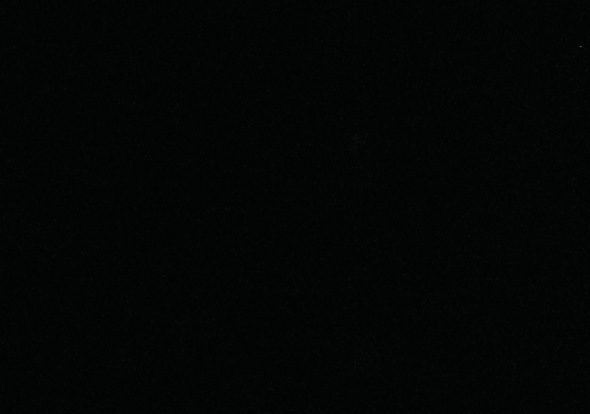
 IFNα

IEP


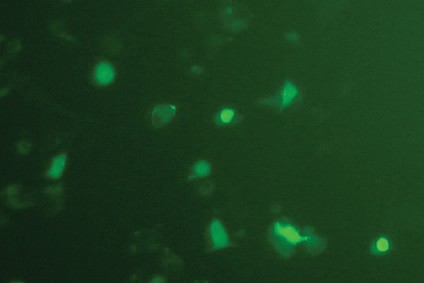


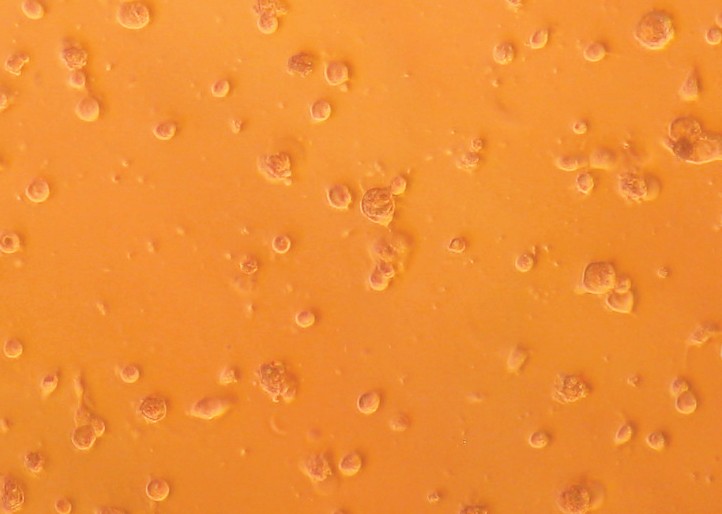


600


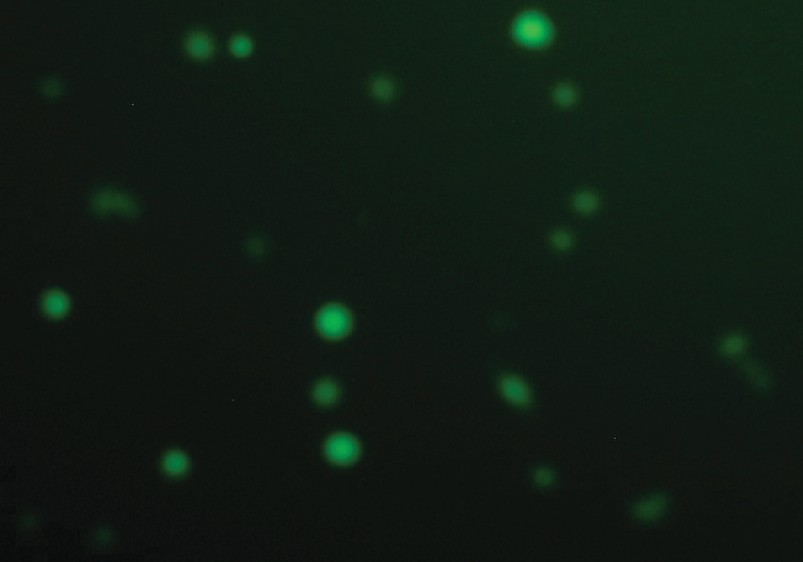


500

IFNα secretion (pg/ml)

400

300


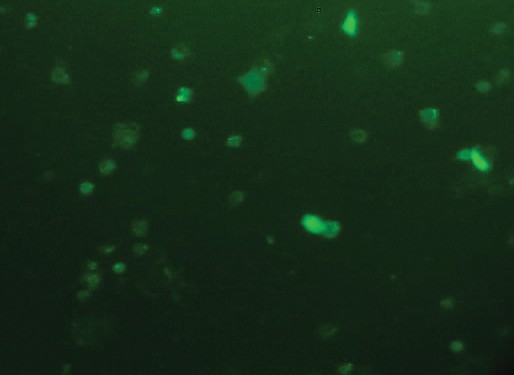


200

100


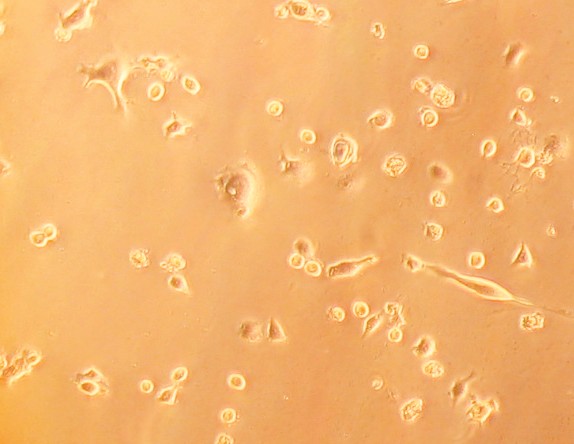


PBS IFNα SIFα IEP

vector *

*

* *

* *

Vector


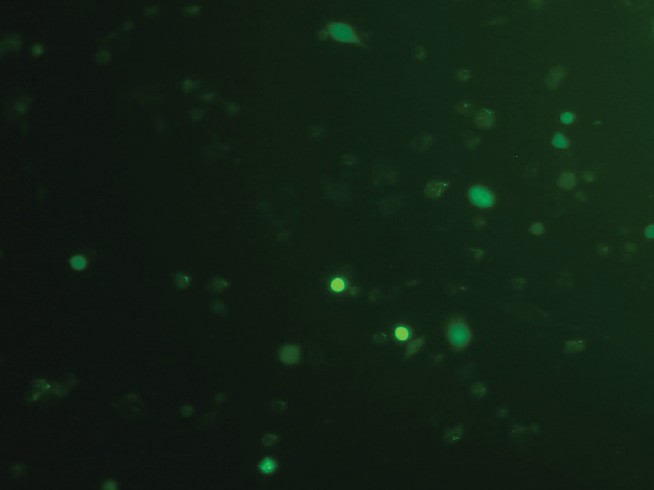


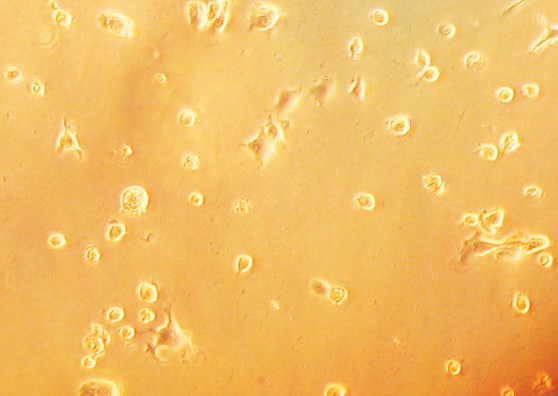


0

24 h 48 h 72 h

**Figure S2. Secretion of synthetic interferon SIF**α

A. Tracking viral infection by copGFP fluorescence in CFPAC1 cells.

B. Quantitation of the secreted interferons in cell supernatants of CFPAC1 cells. After lentiviral infection, the ASPC cell supernatants were collected at three time points and the secreted interferons were measured by ELISA. * p<0.01 as compared with PBS, SP, and vector controls. There is not statistically significant difference between the IFNα and SIFα groups.

C. Viral infection by copGFP fluorescence in ASPC cells.

D. Quantitation of the secreted interferons by ELISA in cell supernatants of ASPC

cells. * p<0.01 as compared with PBS, SP, and vector controls.

Supplementary Figure S3


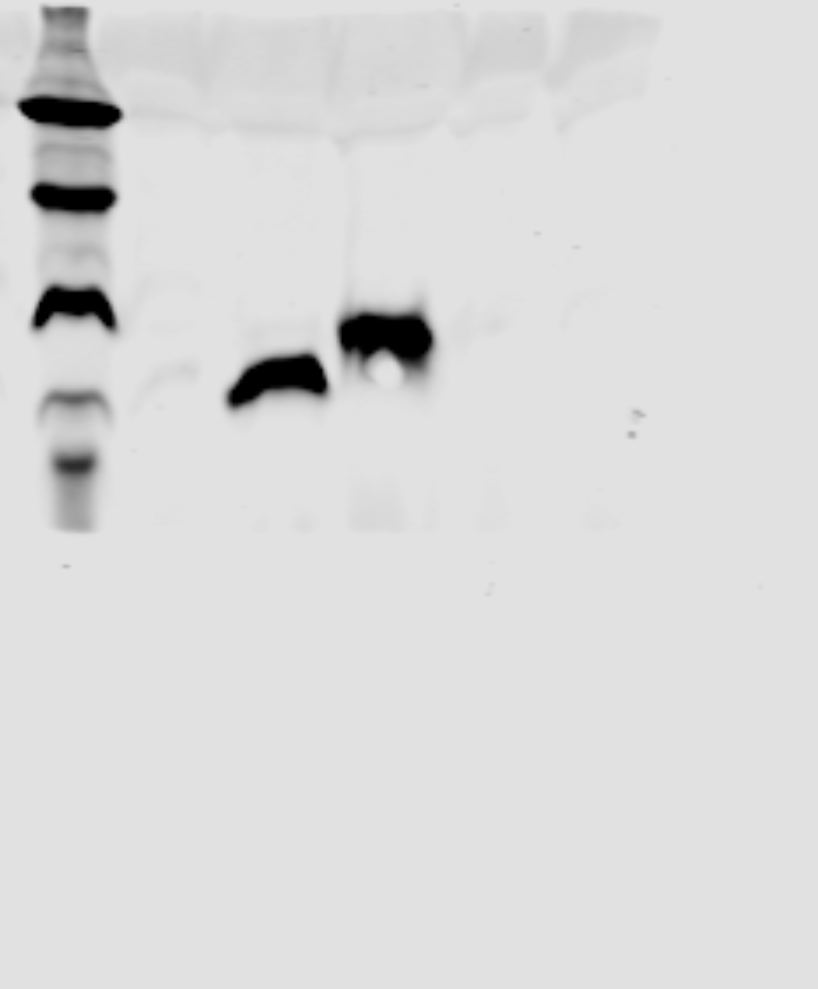


Figure S3. Original picture of Western blot.

(lane 1: PBS, lane 2: IFNα, lanes 3: SIFα, lane 4: IEP,

lane 5: vector)

Supplementary Figure S4

Marker PBS IFNα SIFα IEP vector


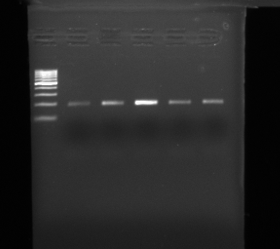
 OAS2 (186 bp)

Figure S4. Original pictures of PCR agarose gels (OAS2)

Supplementary Figure S5

Marker PBS IFNα SIFα IEP vector


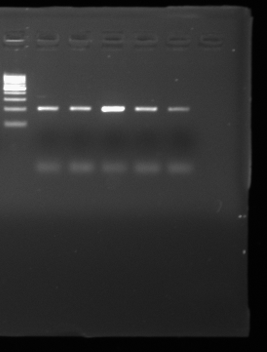
 MX1 (199 bp)

Figure S5. Original pictures of PCR agarose gels (MX1)

Supplementary Figure S6

Marker PBS IFNα SIFα IEP vector


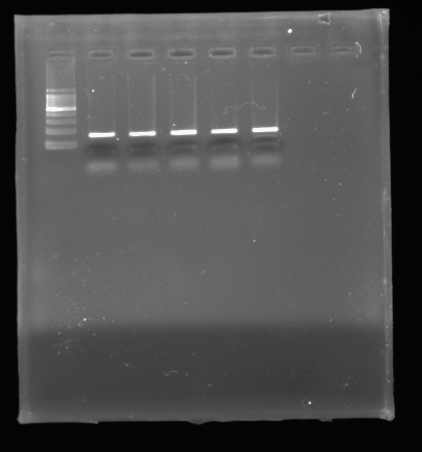
 ADPR (160 bp)

Figure S6. Original pictures of PCR agarose gels (ADPR)

Supplementary Figure S7

vector IEP SIFα IFNα PBS Marker


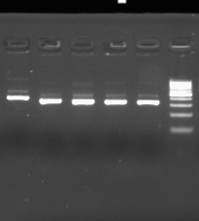
 IFIT1 (329bp)

Figure S7. Original pictures of PCR agarose gels (IFIT1)

Supplementary Figure S8

Marker PBS IFNα SIFα IEP vector


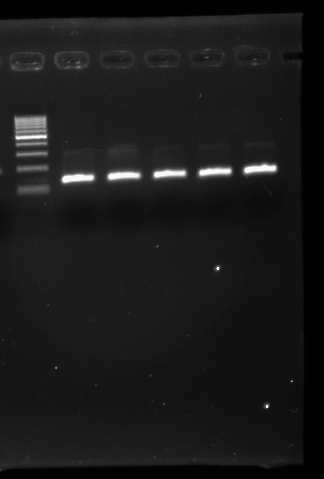
 β-ACTIN (135bp )

Figure S8. Original pictures of PCR agarose gels (β-ACTIN)
